# Family Members’ Feedback on the “Quality of Death” of Adult Patients Who Died in Intensive Care Units and the Factors Affecting the Death Quality: A Systematic Review and Meta-Analysis

**DOI:** 10.7759/cureus.58344

**Published:** 2024-04-15

**Authors:** Kazuaki Naya, Hideaki Sakuramoto, Gen Aikawa, Akira Ouchi, Shun Yoshihara, Yuma Ota, Saiko Okamoto, Ayako Fukushima, Haruyoshi Hirashima

**Affiliations:** 1 Department of Adult Nursing, Tokyo Healthcare University Wakayama Faculty of Nursing, Wakayama, JPN; 2 Department of Critical Care and Disaster Nursing, Japanese Red Cross Kyushu International College of Nursing, Munakata, JPN; 3 Department of Adult Health Nursing, College of Nursing, Ibaraki Christian University, Hitachi, JPN; 4 Department of Fundamental Nursing, Tokyo Healthcare University Faculty of Healthcare, Shinagawa, JPN; 5 Department of Nursing, Hitachi General Hospital, Hitachi, JPN; 6 Advanced Critical Care Center, Kurume University Hospital, Kurume, JPN

**Keywords:** death quality, bereaved family, systematic review, intensive care unit (icu), quality of dying and death

## Abstract

Intensive care units (ICUs) are designed for critically ill patients who often experience high mortality rates owing to the severity of their conditions. Although the primary goal is patient recovery, it is crucial to understand the quality of death in the ICU setting. Nevertheless, there is a notable lack of systematic reviews on measured death quality and its associated factors. This study aims to conduct a quantitative synthesis of evidence regarding the quality of death in the ICU and offers a comprehensive overview of the factors influencing this quality, including its relationship with the post-intensive care syndrome-family (PICS-F). A thorough search without any language restrictions across MEDLINE, CINAHL, PsycINFO, and Igaku Chuo Zasshi databases identified relevant studies published until September 2023. We aggregated the results regarding the quality of death care for patients who died in the ICU across each measurement tool and calculated the point estimates and 95% confidence intervals. The quantitative synthesis encompassed 19 studies, wherein the Quality of Dying and Death-single item (QODD-1) was reported in 13 instances (Point estimate: 7.0, 95% CI: 6.93-7.06). Patient demographic data, including age and gender, as well as the presence or absence of invasive procedures, such as life support devices and cardiopulmonary resuscitation, along with the management of pain and physical symptoms, were found to be associated with a high quality of death. Only one study reported an association between quality of death and PICS-F scores; however, no significant association was identified. The QODD-1 scale emerged as a frequently referenced and valuable metric for evaluating the quality of death in the ICU, and factors associated with the quality of ICU death were identified. However, research gaps persist, particularly regarding the variations in the quality of ICU deaths based on cultural backgrounds and healthcare systems. This review contributes to a better understanding of the quality of death in the ICU and emphasises the need for comprehensive research in this critical healthcare domain.

## Introduction and background

The intensive care unit (ICU) is primarily designed to admit severely ill patients with the potential for recovery. However, high severity often corresponds to a high mortality rate. According to the 2021 report from the Japanese Society of Intensive Care Medicine, based on the Japan ICU Patient Database, the mortality rate at the time of ICU discharge for emergency admissions, excluding scheduled admissions (31.1%), was 6.1% [1]. Additionally, with the anticipated increase in the elderly population [2], the number of patients with high mortality risk is predicted to continue to rise.

However, providing adequate end-of-life care in the ICU remains challenging [3], and a significant number of healthcare professionals involved in intensive care experience difficulties in end-of-life care in the ICU setting [4, 5]. Moreover, given that being a family member of a nonviable patient is identified as a risk factor for the occurrence of post-intensive care syndrome-family (PICS-F) in families of ICU patients [6], end-of-life care in ICUs may be considered inadequate and might not alleviate the psychological burden on the family. However, the influence of the quality of death on PICS-F remains unclear.

A growing body of research exploring the quality of dying and death in the ICU has investigated various evaluation indicators that have been used in this study [7-9]. Despite the importance of understanding the quality of death and dying in ICU patients, to our knowledge, no systematic review has addressed the measure of the quality of dying and the factors related to that quality.

Therefore, we conducted this review to provide a quantitative synthesis of the evidence regarding the quality of deaths in the ICU and to offer a comprehensive overview of the factors influencing this quality, including its relationship with PICS-F.

## Review

Materials and methods

Protocol and Registration

We conducted this systematic review and meta-analysis according to the recommendations of the Preferred Reporting Items for Systematic Reviews and Meta-Analyses (PRISMA) (see Appendices) [10]. The systematic review protocol was registered in the International Prospective Register of Systematic Reviews (registration number: CRD42023466190).

Literature search strategy

The following databases were searched from inception to September 27, 2023: MEDLINE (via PubMed), CINAHL (via EBSCOhost), PsycINFO (via Ovid), and Igaku Chuo Zasshi (see Appendices). We also scanned the World Health Organization International Clinical Trials Registry Platform for ongoing trials. In addition, we conducted citation research (based on Google Scholar) of the identified studies from the period after the second screening to November 5, 2023. Language restrictions were not imposed.

Study screening and selection

Two of the nine reviewers independently screened the titles and abstracts of all studies to identify potentially relevant studies. Subsequently, the full text of these articles was independently reviewed according to a standardised protocol. Disagreements between the two reviewers were resolved through discussion, and if necessary, a third person was brought in for arbitration.

Eligibility Criteria

The inclusion criteria were as follows: (1) study participants must be families of adult patients who had died in the ICU; (2) randomised controlled trials, cross-sectional studies, cohort studies, quantitative studies, and mixed methods studies in which the quality of dying and death for patients who had died in the ICU was evaluated; and (3) no language restriction were applied. The exclusion criteria were as follows: qualitative studies, case studies, letters, commentary papers, review studies, books, conference abstracts, and papers published in non-academic journals.

Main Outcome

This review included studies describing the quality of death as perceived by the loved ones of adult patients who had passed away in the ICU, factors related to the quality, and the association between the quality and PICS-F scores. We defined the quality of death in accordance with previous studies [11] as the degree to which a person's preferences for dying and the moment of death align with observations of how the person died, as reported by others. This review summarises the assessment tools that measure the quality of ICU deaths, integrates the findings on the quality of ICU deaths to identify the factors influencing the quality of death, and assesses the association between death quality and PICS-F scores.

Data Extraction and Quality Assessment

Two of the nine reviewers independently extracted the study data, which included the authors’ names, year of publication, study design, country, survey period, measurement tools used, and characteristics of patients and responders. Outcome data encompassed the quality of death, factors influencing the quality, and the association between the quality of death and PICS-F. We contacted the authors of papers lacking details about the quality of death to obtain the necessary data. In cases of multiple publications for a single study, the reports were grouped, and publications with the most complete data were used for the analysis. Further, two of the nine reviewers independently appraised the studies during the data extraction phase using the Mixed Methods Assessment Tool (MMAT) [12]. The studies were assessed using a categorical scale as "no," "can't tell," or "yes," to reflect their adherence to the evaluated methodological quality criteria. Methodological ratings were reported for each study as 0, 25, 50, 75, and 100, with 100% representing the highest quality.

Statistical Analysis

Based on the data obtained, we aggregated the quality of death and end-of-life care for patients who died in the ICU across each measurement tool. Point estimates and 95% confidence intervals were calculated. The analysis was conducted using R statistical software version 4.3.0 (R Foundation for Statistical Computing, Vienna, Austria) and the “meta” package.

Results

**Figure 1:**
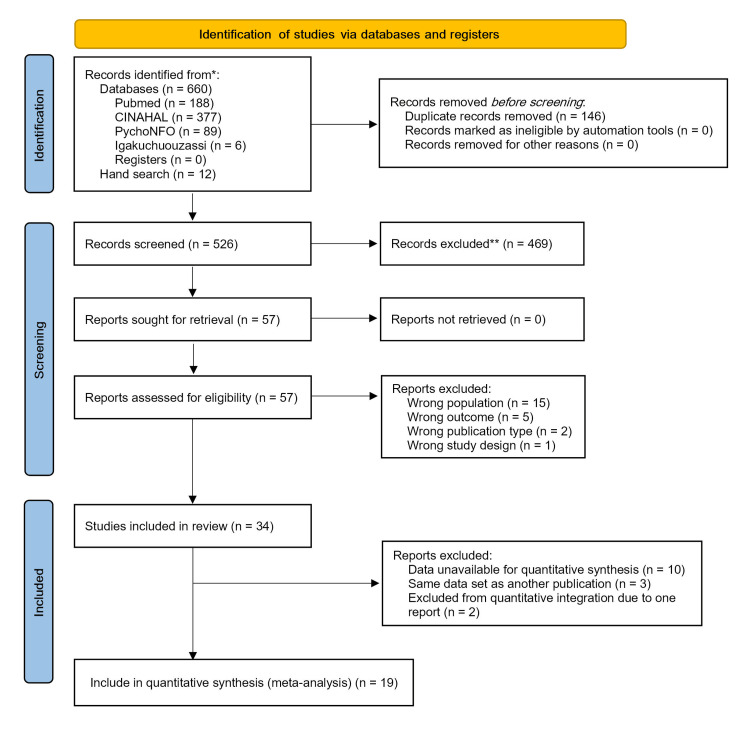
The Preferred Reporting Items for Systematic Reviews and Meta-Analysis 2020 flow diagram

The electronic databases and manual search yielded 672 records (660 from electronic databases and 12 from manual search), and after removing duplicates, 526 papers underwent primary screening. Based on the titles and abstracts, 57 papers that met the eligibility criteria were subjected to secondary screening. After reviewing the full text of these 57 papers, 23 were excluded because they did not meet the eligibility criteria. This resulted in 34 papers selected for review. Among them, 19 papers were included for data integration after excluding 15 papers [8, 9, 13-25] in which data extraction or acquisition was not possible (Figure 1).

There were six intervention studies, comprising four randomised controlled trials [13, 26-28] and two pre-post comparison trials [18, 28]. The remaining 28 studies were observational, and among them, five focused on the development or validation of evaluation scales and translated versions [24, 25, 29-31]. In terms of geographic distribution, most studies were conducted in the American continents (United States: 20, Canada: one, Brazil: three), followed by Europe (Netherlands: five, Denmark: two), and Asia (Japan: two, Korea: one, China: one, Thailand: one). The most frequently used measurement tool was QODD, with variations including single-item versions (QODD-1), versions with different item numbers, and the ICU-version QODD (ICU-QODD) (Table 1).

**Table 1:** Characteristics of the included studies QODD, Quality of dying and death questionnaire; ICU - QODD, ICU version: Quality of dying and death questionnaire; QODD - 1, Single item rating of the overall quality of dying and death; euroQ2, European Quality Questionnaire; FS - ICU, Family Satisfaction with the Intensive care unit; GDI, Good Death Inventory; BFS, Bereaved Family Survey; RCT, Randomized controlled trial; IQR, Inter quartile range; SD, Standard deviation; USA, United States; NZ, New Zealand; DK, Denmark; ED, Emergency department; *PHQ; PCL; HADS; IES*

Authors, Year Country	Study design	Measurement tool and Survey Period (after death)	Patients age [median (IQR) or mean (SD)], Female: n (%)	ICU days [median (IQR) or mean (SD)]	Respondents age [median (IQR) or mean (SD)], Female: n (%)	Relationship to patient
Wen FH, et al., 2023 [7], Taiwan	Prospective cohort study	ICU - QODD (23 items), approximately 1 month	66.51 ± 14.18, 113 (36.6%)	21.2 ± 15.17	49.83 ± 12.55, 184 (59.5%)	Spouse: 91 (29.4%) Child: 166 (53.7%)
Neville TH, et al., 2023 [8], USA	Prospective cohort study	BFS, 3 months	Intervention (I): 69 (57 － 75), 64 (54.7%) Usual care (u): 70 (61 － 79), 83 (42.1%)	Hospital length of stay I: 14 (5 － 25) U: 7 (2 － 17)	Unclear	Unclear
Koyauchi T, et al., 2022 [9], Japan	Observational study	GDI, Median: 24 months (IQR: 17 － 34)				
Kross EK, et al., 2012 [13], USA	Part of cluster - RCT	QODD (31 item ver.) and FS – ICU, Median: 36 days (IQR: 31 - 76.5)	71.0 ± 14.4, 452 (41.9%)	3 days (1 － 7)	58.5 ± 14.6, 749 (69.4%)	Spouse/partner: 485 (44.9%) Child: 385 (35.7%)
Long AC, et al., 2014 [14], USA	Cohort study	QODD (31 items), 4 to 6 weeks	Admission from the Ward: 69.3 ± 12.8, 116 (36%) ED: 70.8 ± 14.4, 457 (44%)	Ward: 7.4 ± 10.8 ED: 5,2 ± 7.5	Ward: 58.3 ± 15.0, 111 (67%) ED: 57.8 ± 14.5, 283 (69%)	Spouse/partner, Child Ward: 91 (53%), 56 (32%) ED: 161 (40%), 153 (38%)
Santos MF, et al., 2011 [15], Brazil	Cross-sectional study	QODD (22 item), Unclear	77.2 ± 12.5, 28 (46.7%)	17.5 ± 11.9	51.7 ± 12.1, 49 (81.7%)	Son/Daughter: 9 (15.0%) Consort: 16 (26.7%) Grandchild: 21 (35.0%)
Mularski RA,, et al., 2004 [16], USA	Cross-sectional study	ICU - QODD (23 items), 4 months to 1 year	59 ± 15, 12 (31.6%)	6 (range: 3 － 43)	Unclear	Unclear
O'Mahony S, et al., 2009 [17], USA	Case-control study	ICU - QODD (21 item), unclear	70.3 (SD: unclear), Sex: Unclear	Unclear	Unclear	Unclear
Braus N, et al., 2015 [18], USA	Prospective, Before - after trial	QODD (QODD - 1) and FS - ICU, PHQ - 8, PCL - C 6 weeks	Intervention (I): 64.4 ± 19.0, 51 (49.5%) Control (C): 60.6 ± 16.6, 41 (41%)	14 days or more, n (%) I: 1 (1%), C: 8 (8%)	I: 60.9 ± 13.9, 36 (62.1%) C: 60.2 ± 13.4, 46 (74.2%)	Spouse / Partner, Child I: 24 (42%), 15 (26%) C: 35 (56%), 11 (18%)
Wachterman M, et al., 2016 [19], USA	Cross-sectional study	BFS, unclear				
Rolnick JA, et al., 2020 [20], USA	Prospective cohort study	BFS, 2 to 6 weeks	70.1 ± 11.4, 292 (2.6%)	Unclear	Unclear	Spouse: 4,669 (42%) Child: 3,226 (29%)
Kinoshita S, et al., 2012 [21], Japan	Cross-sectional study	GDI, Within 10 years	Unclear	Unclear		Spouse: 29 (7.5%) Child: 2 (0.5%)
Teno JM, et al., 2005 [22], USA	Mortality follow-back survey	Assessed in five specifics unclear				
ACM Brekelmans, et,al. [23], NZ	Cross-sectional study	euroQ2, Median: 334 days (IQR: 210 － 420)	71 (65 － 76), 42 (44.2%)	3 (1 － 8)	58 ± 12, 61 (64.9%)	Partner: 42 (44.2%) Daughter / Son: 43 (45.3%)
Meneguin S, et al., 2022 [24], Brazil	Methodological study	QODD, 30 to 90 days	41 ± 15, Sex: Unclear	20 ± 17	47 ± 18, 17 (62%)	Child (66%)
Benichel CR, et al., 2023 [25], Brazil	Methodological study	QODD, 30 to 90 days	Age: Unclear, 136 (41.7%)	Unclear	45 ± 13.7, 210 (64.4%)	Spouse/companion: 48 (14.7%) Son/daughter: 177 (54.3%)
Curtis JR, et al., 2011 [26], Canada	Cluster - RCT	QODD (31 item ver.), 4 to 6 weeks	Intervention (I): 71.1 ± 14.3, 242 (47.1%) Control (I): 71.0 ± 14.8, 221 (39.1%)	I: 5.0 ± 6.3 C: 6.0 ± 12.3	I: 58.1 ± 14.5, Female: 63.1% C: 59.3 ± 14.5, 221 (39.1%)	Spouse I: 40% (72), C: 46.9% (98)
Kross EK, et al., 2014 [27], USA	Part of cluster - RCT	QODD (31 items), 1 to 2 months	69.2 ± 15.2, Female: 41.5%	Unclear	58.2 ± 14.3, Female: 68.2%	Spouse: 45.2%
Khandelwal N, et al., 2014 [28], USA	Before - after trial, Cluster - RCT	QODD (QODD - 1) and FS – ICU, 4 to 6 weeks	63 ± 17.7, 229 (37.7%)	3 (1 － 8)	57 ± 17.7, 199 (64.8%)	Spouse: 40%
Han XP, et al., 2021 [29], Chaina	Validation study	ICU - QODD (22 item), Unclear	Age group, n (%) ≦ 30: 16 (11%), 31 － 50: 38 (25%) 51 － 70: 58 (39%), > 70: 37 (25%) Female: 45 (30%)	≦ 3 days: 98 (66%) 4 － 7 days: 32 (21%) > 7 days: 19 (13%)	Age group, n (%) ≦ 30: 20 (16%) 31 － 50: 102 (69%) > 50: 23 (15%) Female: 57 (38%)	Spouse: 43 (29%) Offspring: 81 (54%)
Jensen HI, et al., 2015 [30], DK and NZ	Two site cross-sectional survey	euroQ2, HADS, and IES – R, 3 weeks	DK: 74 (65 － 79), 15 (38.5%) NZ: 70 (61 － 76), 16 (38.1%)	DK: 7.5 (5.2 － 15.0) NZ: 6.8 (54.1 － 10.3)	DK: 57 ± 13, 30 (54.5%) NZ: 52 ± 14, 35 (63.6%)	Partner, Daughter / son DK: 27 (49%), 22 (40) NZ: 21 (38%), 28 (51%)
Gerritsen RT, et al., 2018 [31], DK and NZ	Usability study, Prospective study	euroQ2, 3 weeks	70.8 ± 10.5, 72 (41.4%)	8.2 (IQR: 12.0)	56.1 ± 14.0, 137 (64.6%)	Spouse/partner: 79 (37.1%) Child: 99 (46.5%)
Choi Y, et al., 2019 [32], Korean	Retrospective study	QODD (17 item), Unclear	82 (69-87), 4 (25%)	17.5 (range: 5 － 46)	52 (32 － 75), 16 (100%)	Spouse: 1 (6.3%) Adult child: 15 (93.7%)
Mularski RA,, et al., 2004 [33], USA	Prospective cohort study	ICU - QODD (23 items) and QODD (31 items), 4 months	59 ± 15, Female: 29%	11 (range: 3 － 43)	47 ± 14, 67 (71.3%)	Spouse/partner: 18 (19%) Child: 27 (29%)
Gerritsen RT, et al., 2017 [34], USA	Prospective cohort study	ICU - QODD (23 items), 4 to 6 weeks	66 ± 15.2, 160 (35.9%)	6 (3 － 13)	57 ± 14.3, Sex: Unclear	Unclear
Curtis JR,, et al., 2008 [35], USA	Cross-sectional study	ICU - QODD (21 items), 4 to 6 weeks	Preintervention (Pre): 62.0 ± 17.51, 85 (33.6%) Postintervention (Post): 61.7 ± 17.65, 115 (34.1%)	Pre: 3.85 (1.57 － 9.47) Post: 3.06 (1.02 － 7.25)	Pre: 56.0 ± 12.95, 77 (61.6%) Post: 56.9 ± 14.45, 120 (80.0%)	Spouse, Child Pre: 44.0 (55%), 31.2 (39%) Post: 45.3 (68%), 22.7 (34%)
Lewis-Newby M, et al., 2011 [36], USA	Prospective study	ICU - QODD (21 items), 4 to 6 weeks	Age < 35: 25.38 ± 5.8, 5 (19.2%) Age 35 － 64: 53.6 ± 6.8, 46 (40.7%) Age ≧ 65: 76.9 ± 7.0, 48 (35.3%)	Age < 35: 3.9 (2.2 － 6.9) Age 35 － 64: 4.8 (1.8 － 9.7) Age ≧ 65: 3.2 (1.5 － 9.9)	Age < 35: 49.0 ± 10.1, 20 (76.9%) Age 35 － 64: 52.5 ± 12.8, 71 (63.4%) Age ≧ 65: 61,22 ± 13.6, 88 (65.7%)	Spouse / Partner, Child Age < 35: 2 (7.7%), 0 (0%) Age 35 － 64: 55 (48.7%), 18 (15.9%) Age ≧ 65: 64 (47.4%), 55 (40.7%)
Levy CR, et al., 2004 [37], USA	Cross-sectional study	ICU - QODD (21 items), 1 month	64 ± 14.5, 33 (48.5%)	6.92 ± 5.99	Unclear	Unclear
Glavan BJ, et al., 2008 [38], USA	Cross-sectional study	ICU - QODD (22 items), 1 to 2 months	70.1 ± 15.9, 147 (41.3%)	2.8 (0.9 － 7.1)	Unclear	Spouse / partner: 145 (42.6%) Child: 118 (34.7%)
Lee JJ, et al., 2016 [39], USA	Observational study	QODD (QODD - 1), 3 to 5 weeks	Nonminority (N): 70.8 ± 14.5, 460 (41.3%) Minority (M): 64.5 ± 17.1, 68 (38.6%)	Unclear	N: 59.3 ± 14.2, 719 (67.6%) M: 50.9 ± 14.7, 132 (72.9%)	Spouse N: 508 (47.7%), M: 63 (34.8%)
Osborn TR, et al., 2012 [40], USA	Multisite cross-section	QODD (QODD - 1) and FS – ICU, 4 to 6 weeks	69.9 (15), 528 (41.8%)	unclear	58.0 ± 14.5, 862 (70.9%)	Spouse / partner: 577 (45.6%) Adult child: 444 (35.1%)
DeCato TW, et al., 2013 [41], USA	Retrospective cohort	QODD (QODD - 1) 4 to 6 weeks	69.1 ± 15.7, Female: 41.4%	5.7 ± 9.0	58.2 ± 14.5, 1187 (68.5%)	Unclear
Gerritsen RT, et al., 2013 [42], NZ	Prospective study	QODD (QODD - 1) 3 to 4 weeks	73 (65 － 80), 34 (34%)	8 (3 － 16)	Unclear	Partner: 31 (31%) Child: 68 (68%)
Vattanavanit V, et al., 2017 [43], Thailand	Cross-sectional study	QODD (QODD - 1), 1 month	Buddhist (B): 61.8 ± 16.7, 54 (60.4%) Muslim (M): 54.3 ± 13.8, 10 (47.6%)	Buddhis: 3 (1 － 9) Muslim: 2 (1 － 7)	B: 44.6 ± 10.3, 69 (75.8%) M: 42.7 ± 10.8, 12 (57.1%)	Unclear

Quality Assessment

Among the studies included for quantitative synthesis, 16 met 80% of the quality criteria and three met 60% of the quality criteria. However, most studies focused on specific regions or populations, and the data were not considered representative of the general population (Table 2).

**Table 2:** Quality assessment of included studies for quantitative synthesis using MMAT MMAT = Mixed Methods Appraisal Tool; Y = yes; N = no; U = can’t tell All studies going through quality assessment passed the screening questions: 1) Are there clear research questions? 2) Do the collected data allow us to address the research questions? (Methodological quality criteria) 2.1. Is randomization appropriately performed? 2.2. Are the groups comparable at baseline? 2.3. Are there complete outcome data? 2.4. Are outcome assessors blinded to the intervention provided? 2.5 Did the participants adhere to the assigned intervention? 3.1. Are the participants representative of the target population? 3.2. Are measurements appropriate regarding both the outcome and intervention (or exposure)? 3.3. Are there complete outcome data? 3.4. Are the confounders accounted for in the design and analysis? 3.5. During the study period, is the intervention administered (or exposure occurred) as intended? 4.1. Is the sampling strategy relevant to address the research question? 4.2. Is the sample representative of the target population? 4.3. Are the measurements appropriate? 4.4. Is the risk of nonresponse bias low? 4.5. Is the statistical analysis appropriate to answer the research question?

2. Quantitative randomized controlled trials	Authors, year	Methodological quality criteria					
		2.1	2.2	2.3	2.4	2.5	Score (%)
	Curtis JR, et al., 2011 [26]	N	Y	N	Y	Y	60
3. Quantitative nonrandomized	Authors, year	Methodological quality criteria					
		3.1	3.2	3.3	3.4	3.5	Score (%)
	Wen FH, et al., 2023 [7]	Y	Y	Y	N	Y	80
	Curtis JR,, et al., 2008 [35]	Y	Y	Y	N	Y	80
	Lewis-Newby M, et al., 2011 [36]	Y	Y	Y	N	Y	80
	Levy CR, et al., 2004 [37]	Y	Y	Y	N	Y	80
	Glavan BJ, et al., 2008 [38]	Y	Y	Y	N	Y	80
4. Quantitative descriptive	Authors, year	Methodological quality criteria					
		4.1.	4.2.	4.3.	4.4.	4.5.	Score (%)
	Kross EK, et al., 2014 [27]	Y	Y	Y	N	Y	80
	Khandelwal N, et al., 2014 [28]	Y	Y	Y	N	Y	80
	Han XP, et al., 2021 [29]	Y	Y	N	Y	Y	80
	Jensen HI, et al., 2015 [30]	Y	Y	Y	N	Y	80
	Gerritsen RT, et al., 2018 [31]	Y	Y	Y	N	Y	80
	Choi Y, et al., 2019 [32]	Y	Y	N	N	Y	60
	Mularski RA,, et al., 2004 [33]	Y	Y	Y	Y	Y	100
	Gerritsen RT, et al., 2017 [34]	Y	N	Y	N	Y	60
	Lee JJ, et al., 2016 [39]	N	Y	Y	Y	Y	80
	Osborn TR, et al., 2012 [40]	Y	Y	Y	N	Y	80
	DeCato TW, et al., 2013 [41]	Y	Y	Y	Y	Y	100
	Gerritsen RT, et al., 2013 [42]	Y	Y	Y	Y	Y	100
	Vattanavanit V, et al., 2017 [43]	Y	Y	Y	Y	Y	100

Quantitative Synthesis of Quality of Death in the ICU

Various instruments, including the QODD, ICU-QODD, QODD-1, EuroQ2, Bereaved Family Survey (BFS), and Good Death Inventory (GDI) scales were employed to measure the quality of death in the ICU. Average scores were aggregated for each measurement tool (Table 3).

**Table 3:** Meta-analysis based on each measurement tool QODD, Quality of dying and death questionnaire; ICU - QODD, ICU version Quality of dying and death questionnaire; QODD - 1, Single item rating of the overall quality of dying and death; euroQ2, European Quality Questionnaire

Valuation indicator	Studies (n)	Total sample size	Point estimate	Lower 95% CI	Upper 95% CI
QODD (Score range: 0 － 100)	3	1349	60.5	59.31	61.70
ICU – QODD (Score range: 0 － 100)	6	1345	64.0	62.71	65.22
QODD – 1 (Score range: 0 － 10)	13	7918	7.0	6.93	7.06
euro Q2 (Overall rating of care) (Score range: 0 － 10)	2	307	9.1	8.98	9.21

Although the QODD total score was reported in six studies [13, 14, 16, 26, 32, 33], the presence of overlapping datasets necessitated the selection of papers with the most comprehensive data for the integration of QODD total scores [26, 32, 33]. For studies utilising the ICU-QODD with the same dataset [16, 33], the paper with the most complete data was selected [33] and integrated with the average ICU-QODD total scores [33-38]. Among the studies using QODD or ICU-QODD, 13 were identified from which QODD-1 scores could be extracted. The average scores were integrated for these studies [7, 26-29, 34, 36, 38-43].

Concerning EuroQ2, the overall rating of care [30, 31] and the total score [23] were reported. However, as only one study reported the total score, the average score was integrated into the overall rating of care.

Of the three studies using the BFS, data could not be obtained exclusively for patients who died in the ICU [19], and for another study, the BFS measurement scores could not be retrieved [8]. Consequently, only one study with extractable measurement scores [overall rating of care was excellent: 56.6 (55.1-58.1)] was included [20]. We were unable to obtain measurement scores for two studies utilising the GDI [9, 21].

Factors Influencing the Quality of Death

Eleven studies reported factors related to the quality of death (Table 4). Of these, 10 were observational studies [7, 14, 16, 20, 30, 32, 36, 38-40] and one was an intervention study [28]. In terms of patient demographics, older age, and male gender were associated with a higher quality of dying [36, 38, 39], while being a member of a socially marginalised racial or ethnic group was associated with lower quality of dying [39]. Regarding the ICU treatment environment and conditions, factors such as dying in a state with fewer life-sustaining devices and the absence of cardiopulmonary resuscitation, along with prior articulation of patient preferences for withdrawal from life-sustaining devices, were associated with a higher quality of dying [38, 39]. Entering the ICU from a general ward and subsequently dying in the ICU was associated with a lower quality of dying, but longer ICU stay was positively correlated with family evaluations of care in the ICU [14, 20]. Additionally, high family satisfaction with ICU care was associated with the quality of dying [7, 30]. Factors associated with higher quality of dying were as follows: (1) effective management of patient pain and physical symptoms [7, 16, 38]; (2) the ability to control the surrounding environment, including the degree to which the patient and family are presented with a variety of options, understand them, and feel in control of their choices [16, 40]; and (3) maintenance of the patient's self-respect and dignity [16, 40].

**Table 4:** Factors associated with quality of death QODD, Quality of dying and death questionnaire; ICU - QODD, ICU version Quality of dying and death questionnaire; QODD-1, Single item rating of the overall quality of dying and death; euroQ2, European Quality Questionnaire; FS - ICU, Family Satisfaction with the Intensive care unit; BFS, Brief Fatigue Inventory; β, Standardized Estimate; B, Parameter Estimate

Authors, Year	Instrument Used	Associations With Overall quality of dying and death	
		Positive	Negative or Neutral
Long AC, et al., 2014 [14]	QODD - 1		Patients admitted to the ICU from hospital wards. QODD-1 Ratings average Ward: 6.5 vs ED: 7.3 (P = 0.006, 95% CI: - 1.41, - 0.24)
Lee JJ, et al., 2016 [39]	QODD - 1	Path analysis of factors influencing of the quality of dying and death: 1) Death in the absence of full support (β = - 0.812, P < 0.001) 2) Older patients (irrespective of their living will and life-support status) (β = 0.016, P < 0.01)	Minority family members QODD-1 Ratings average Minority: 7.1 ± 3.0 vs Nonminority: 6.1 ± 3.6 (P < 0.001)
Rolnick JA, et al., 2020 [20]	BFS	For patients receiving mixed care, increased time in the ICU was associated with higher adjusted family ratings of care.	Mixed care with death in the ICU
Wen FH, et al., 2023 [7]	QODD - 1 FS-ICU	Patients in the high QODD class had optimal physical symptom control, moderate-sufficient emotional preparedness for death and few Life-sustaining treatments received.	Bereaved surrogates in the worst QODD class scored lowest in the FS - ICU Care and FS - ICU Decision- Making subscales.
Mularski RA,, et al., 2004 [16]	ICU - QODD	Multivariate Model Exploring Associations to QODD Rating Score: 1) Feeling at peace with dying (B = 3.84, SE = 0.69, 95% CI = 2.49, 5.19, P < 0.0001) 2) Pain under control (B = 3.82, SE = 0.88, 95% CI = 2.09, 5.56, P < 0.0001) 3) Control of events (B = 2.17, SE = 0.80, 95% CI = 0.60, 3.73, P = 0.0066) 4) Keep dignity/self-respect (B = 2.02, SE = 0.67, 95% CI = 0.69, 3.34, P = 0.0028)	
Glavan BJ, et al., 2004 [38]	QODD QODD - 1	Patient age: Increasing age, Patient sex: Male Significant independent predictors of high scores on the QODD - 22 were: 1) Family presence at time of death 2) Documentation of the patient's desire to be weaned off life support at a family meeting 3) Documentation of pain assessment 4) no cardiopulmonary resuscitation at the end of life	
Lewis-Newby M, et al., 2011 [36]	QODD QODD - 1	Patient age: Increasing age (Age group: Age < 35, Age 35 - 64, Age ≧ 65) QODD (median, IQR): 57.6 (50.8 － 78.8), 65.6 (45.2 － 82.7), 72.9 (54.8 － 89.5) QODD - 1 (median, IQR): 8 (5 － 9), 8 (5 － 9.75), 9 (7 － 10)	
Osborn TR, et al., 2012 [40]	QODD - 1	Associations Between FS - ICU Items and QODD - 1 Score 1) support of family as decision-maker (β = 0.10, t = 2.20, P = 0.03) 2) family control over patient’s care (β = 0.18, t = 3.91, P < 0.01) 3) ICU atmosphere (β = 0.12, t = 2.22, P = 0.03)	
Khandelwal N, et al., 2014 [28]	QODD - 1 FS-ICU	For underinsured patients, we found strong evidence of a positive association between the family-assessed QODD - 1 and average daily ICU costs (β = 1.4, 95% CI = 0.4, 2.3, P = 0.01).	
Jensen HI, et al., 2015 [30]	euroQ2	The euroQODD was significantly correlated with the euroFS - ICU (r = 0.54, P = 0.003).	
Choi Y, et al., 2019 [32]	QODD	Transferred out of the ICU, died in a general ward bed. (vs died in the ICU: median, 64.5 vs 45.0, P = 0.095).	

Association Between Quality of Death and PICS-F Scores

One study addressed the relationship between the quality of dying in the ICU and PICS-F scores, which utilised EuroQ2, the Hospital Anxiety and Depression Scale (HADS), and the Impact of Event Scale-Revised (IES-R) [30]. The results of a family survey conducted three weeks after bereavement revealed that 21% of the respondents experienced moderate to severe anxiety symptoms and 10% reported moderate to severe depressive symptoms. However, no significant correlations were observed between EuroQ2 and Family Satisfaction in the ICU composite score or between the Euro-QODD and HADS or IES-R scores.

Discussion

In this review, a quantitative synthesis was performed based on 19 eligible papers that met the criteria and allowed for data extraction. As outcomes, point estimates for the quality of death in the ICU and factors influencing improvement or deterioration in the quality of death were elucidated. However, the association between the quality of death and PICS-F remains unclear.

Quality of Death in the ICU

The QODD has been employed as a metric to assess the quality of death in ICUs, with QODD-1 being the most frequently cited tool. We calculated a point estimate of 7.0 (95% CI: 6.93, 7.06). The QODD-1 is a single-item rating on a visual analog scale, utilising an 11-point scale ranging from 0 = “terrible” to 10 = “almost perfect” on the question “Overall, how would you rate the quality of your loved one's dying?” However, specific cutoff values have not yet been established. Given that in a survey of bereaved families of non-ICU patients, the measured quality of death was 5.82 (SD = 2.72) for patients without Palliative Care Team (PCT) consultation (N = 98) and 6.68 (SD: 2.64) for those with PCT consultation (N = 77) [44], the score of 7.0 (95% CI: 6.93, 7.06) in this review is high. Surveys of survivors of ICU patient deaths [13] have indicated an association between higher survey burden and lower assessed quality of death. In our review, 10 of the 13 studies integrated into the QODD-1 quantitative synthesis were conducted through mail surveys [26-28, 34-36, 38-41]. However, this approach may result in biased outcomes because no responses were received from survivors experiencing a survey burden, potentially leading to inflated point estimates. The QODD has various versions, including those with different number of items (17 and 31 items) and an ICU-specific version. However, the QODD-1, distinguished by its single-item format and efficiency, is particularly well suited for surveys targeting bereaved families facing a high psychological burden. Additionally, considering the correlation observed between the total QODD score and QODD-1 [38], QODD-1 has emerged as a highly recommended tool for assessing the quality of end-of-life care in the ICU.

Factors Associated With the Quality of Death

Factors associated with the quality of death in the ICU comprised "patient background," "ICU treatment environment and conditions," and "management of patient symptoms and maintenance of dignity." Screening for factors potentially influencing patient background is imperative to enhance the quality of death in the ICU. Additionally, interventions aimed at improving factors associated with the "ICU therapeutic environment and conditions" and "management of patient symptoms and maintenance of dignity" are as crucial. Screening items such as "patient is young," "patient is female," "patient is a social/ racial/ethnic minority," "patient entered from a general hospital bed," "availability of adequate mechanical support," and "patient has no advance directives" were identified as contributors to lower death quality. Ensuring the presence of advance directives and understanding the patients’ presumed wishes, particularly in end-of-life care, are essential. Constraints on a patient's life expectancy owing to challenging situations during the dying process can affect the quality of death [11, 45]. The acute and severe onset of ICU disease often necessitates invasive treatment and care, posing challenges to respecting the values and wishes of the patient. Therefore, it is crucial to confirm patients’ wishes and advance directives beforehand to ensure a high quality of death. Factors related to the "treatment environment and conditions in the ICU" and the "management of patient symptoms and maintenance of patient dignity" indicated that effective pain and physical symptom management, preservation of patient self-esteem, and death under less invasive conditions, such as avoiding life-prolonging devices and CPR, were associated with a higher quality of death. In conclusion, the ICU can be deemed a suitable place for patients to spend their final days, aiming to provide comfort during this critical period. The overarching goal is to comfort patients who spend their final hours in the ICU.

Association Between Quality of Dying and PICS-F

The prevalence of anxiety and depression symptoms was reported in a family survey conducted 3 weeks after bereavement (21% for moderate to severe anxiety symptoms and 10% for moderate to severe depression symptoms) [30]. However, this prevalence was not as high as the prevalence of depressive symptoms observed 3 months after bereavement among families of cancer patients (Proportion of clinically meaningful scores on the Center for Epidemiologic Studies Depression Scale: 34%) [46]. Additionally, there was no association between quality of death and PICS-F. However, these results were only observed in one study [30], and the time of the study was 3 weeks after bereavement. Therefore, it is necessary to investigate psychiatric symptoms after 6 months of bereavement, taking into account complicated grief, and to examine the relationship between QODD and PICS-F.

Limitations and implications

Study Limitations

Despite the significant findings, we acknowledge certain limitations of our study. First, methodological heterogeneity due to the variety of survey methods (mail, telephone, or face-to-face interviews) may have affected the results. A study of bereaved families of ICU patients [13] indicated that a high survey burden, including "emotional responses to the survey" and "logistical issues with survey completion (Language, timing, and more)," was associated with a lower assessed quality of death. In this review, 10 out of the 13 studies included in the QODD-1 quantitative synthesis relied on mail surveys [26-28, 34-36, 38-41], potentially leading to inflated point estimates due to non-responses from survivors with perceived survey burden.

Second, the results may lack generalisability to a broader population, as the sample size per study was not large and the study distribution was biased toward the European region. While some studies have compared the quality of death across countries and religions [34, 43], few have delved into factors that consider cultural backgrounds and healthcare systems. This suggests a limitation in understanding the broader implications of quality of death in the ICU across diverse populations and settings.

Research and Clinical Implications

Patient comfort is crucial for enhancing the quality of death in the ICU, emphasising the need for end-of-life care that respects patients’ wishes. Furthermore, considering the emotional and time-consuming nature of survivor surveys, the QODD-1, which is designed for prompt responses, may prove valuable in assessing the quality of death in clinical practice. However, the limitations of this review, including the small sample sizes of each study and geographical bias, prevented the examination of potential differences in the QODD scores based on cultural backgrounds and healthcare systems. Consequently, there is a pressing need for international studies employing QODDs to explore potential cultural variations. Simultaneously, understanding the association between QODDs and PICS-F could significantly contribute to preventing and mitigating mental health issues among bereaved families in the aftermath of loss. Future research in this direction is essential for a more comprehensive understanding of the factors influencing the quality of ICU deaths.

## Conclusions

QODD-1 has emerged as a frequently referenced and valuable metric to evaluate the quality of ICU deaths. Factors, such as patient background and treatment environment play crucial roles, highlighting the need for targeted interventions. However, research gaps persist, particularly regarding the variations in the quality of ICU deaths based on cultural backgrounds and healthcare systems. Additionally, the relationship between the PICS-F and death quality remains unclear and warrants further investigation in future studies. This study contributes to a better understanding of the quality of death in the ICU and emphasises the need for comprehensive research in this critical healthcare domain.

## Appendices

**Table 5:** The Preferred Reporting Items for Systematic Reviews and Meta-Analysis 2020 Checklist

Section and Topic	Item #	Checklist item	Location where item is reported
TITLE			
Title	1	Identify the report as a systematic review.	P. 1
ABSTRACT			
Abstract	2	See the PRISMA 2020 for Abstracts checklist.	P. 1
INTRODUCTION			
Rationale	3	Describe the rationale for the review in the context of existing knowledge.	P. 1-2
Objectives	4	Provide an explicit statement of the objective(s) or question(s) the review addresses.	P. 2
METHODS			
Eligibility criteria	5	Specify the inclusion and exclusion criteria for the review and how studies were grouped for the syntheses.	P. 2
Information sources	6	Specify all databases, registers, websites, organisations, reference lists and other sources searched or consulted to identify studies. Specify the date when each source was last searched or consulted.	P. 2
Search strategy	7	Present the full search strategies for all databases, registers and websites, including any filters and limits used.	P. 2 Table 5
Selection process	8	Specify the methods used to decide whether a study met the inclusion criteria of the review, including how many reviewers screened each record and each report retrieved, whether they worked independently, and if applicable, details of automation tools used in the process.	P. 2
Data collection process	9	Specify the methods used to collect data from reports, including how many reviewers collected data from each report, whether they worked independently, any processes for obtaining or confirming data from study investigators, and if applicable, details of automation tools used in the process.	P. 2
Data items	10a	List and define all outcomes for which data were sought. Specify whether all results that were compatible with each outcome domain in each study were sought (e.g. for all measures, time points, analyses), and if not, the methods used to decide which results to collect.	P. 2
	10b	List and define all other variables for which data were sought (e.g. participant and intervention characteristics, funding sources). Describe any assumptions made about any missing or unclear information.	P. 2
Study risk of bias assessment	11	Specify the methods used to assess risk of bias in the included studies, including details of the tool(s) used, how many reviewers assessed each study and whether they worked independently, and if applicable, details of automation tools used in the process.	P. 2
Effect measures	12	Specify for each outcome the effect measure(s) (e.g. risk ratio, mean difference) used in the synthesis or presentation of results.	P. 2
Synthesis methods	13a	Describe the processes used to decide which studies were eligible for each synthesis (e.g. tabulating the study intervention characteristics and comparing against the planned groups for each synthesis (item #5)).	P. 2
	13b	Describe any methods required to prepare the data for presentation or synthesis, such as handling of missing summary statistics, or data conversions.	N/A
	13c	Describe any methods used to tabulate or visually display results of individual studies and syntheses.	P. 2
	13d	Describe any methods used to synthesize results and provide a rationale for the choice(s). If meta-analysis was performed, describe the model(s), method(s) to identify the presence and extent of statistical heterogeneity, and software package(s) used.	P. 2
	13e	Describe any methods used to explore possible causes of heterogeneity among study results (e.g. subgroup analysis, meta-regression).	N/A
	13f	Describe any sensitivity analyses conducted to assess robustness of the synthesized results.	N/A
Reporting bias assessment	14	Describe any methods used to assess risk of bias due to missing results in a synthesis (arising from reporting biases).	N/A
Certainty assessment	15	Describe any methods used to assess certainty (or confidence) in the body of evidence for an outcome.	N/A
RESULTS			
Study selection	16a	Describe the results of the search and selection process, from the number of records identified in the search to the number of studies included in the review, ideally using a flow diagram.	Figure 1
	16b	Cite studies that might appear to meet the inclusion criteria, but which were excluded, and explain why they were excluded.	N/A
Study characteristics	17	Cite each included study and present its characteristics.	P. 3 Table 1
Risk of bias in studies	18	Present assessments of risk of bias for each included study.	P. 6 Table 2
Results of individual studies	19	For all outcomes, present, for each study: (a) summary statistics for each group (where appropriate) and (b) an effect estimate and its precision (e.g. confidence/credible interval), ideally using structured tables or plots.	N/A
Results of syntheses	20a	For each synthesis, briefly summarise the characteristics and risk of bias among contributing studies.	N/A
	20b	Present results of all statistical syntheses conducted. If meta-analysis was done, present for each the summary estimate and its precision (e.g. confidence/credible interval) and measures of statistical heterogeneity. If comparing groups, describe the direction of the effect.	P. 7-8 Table 3
	20c	Present results of all investigations of possible causes of heterogeneity among study results.	N/A
	20d	Present results of all sensitivity analyses conducted to assess the robustness of the synthesized results.	N/A
Reporting biases	21	Present assessments of risk of bias due to missing results (arising from reporting biases) for each synthesis assessed.	N/A
Certainty of evidence	22	Present assessments of certainty (or confidence) in the body of evidence for each outcome assessed.	N/A
DISCUSSION			
Discussion	23a	Provide a general interpretation of the results in the context of other evidence.	P. 10
	23b	Discuss any limitations of the evidence included in the review.	P. 10-11
	23c	Discuss any limitations of the review processes used.	P. 10-11
	23d	Discuss implications of the results for practice, policy, and future research.	P. 11
OTHER INFORMATION			
Registration and protocol	24a	Provide registration information for the review, including register name and registration number, or state that the review was not registered.	P. 2
	24b	Indicate where the review protocol can be accessed, or state that a protocol was not prepared.	P. 2
	24c	Describe and explain any amendments to information provided at registration or in the protocol.	N/A
Support	25	Describe sources of financial or non-financial support for the review, and the role of the funders or sponsors in the review.	P. 12
Competing interests	26	Declare any competing interests of review authors.	P. 12
Availability of data, code and other materials	27	Report which of the following are publicly available and where they can be found: template data collection forms; data extracted from included studies; data used for all analyses; analytic code; any other materials used in the review.	N/A

**Table 6:** Search strategy

Database	Search Terms
PubMed	Intensive care units[mh] OR critical care[mh] OR critical illness[mh] OR "ICU"[tiab] OR intensive care unit*[tiab] OR "intensive care"[tiab] OR "critical care"[tiab] OR "critical illness"[tiab] OR "critically ill"[tiab] AND Patients[mh] OR Family[mh] OR patient*[tiab] OR "family"[tiab] OR "families"[tiab] OR family member*[tiab] OR relative*[tiab] AND quality of death*[tiab] OR quality of dying*[tiab] OR good death*[tiab] OR bad death*[tiab] OR "QODD"[tiab] OR "GDI"[tiab] OR "CES"[tiab] NOT animals [mh] NOT humans [mh]
CINAHL via EBSCOhost	(MH Intensive care units) OR (MH critical care) OR (MH critical illness) OR ("ICU") OR (intensive care unit*) OR ("intensive care") OR ("critical care") OR ("critical illness") OR ("critically ill") AND (MH Patients) OR (MH Family) OR (patient*) OR ("family") OR ("families") OR (family member*) OR (relative*) AND (quality of death*) OR (quality of dying*) OR (good death*) OR (bad death*) OR ("QODD") OR ("GDI") OR ("CES") NOT (MH animals) NOT (MH humans)
PsycINFO	(MJ Intensive care) OR (MJ Critical Illness) OR (TI intensive care unit*) OR (TI "intensive care") OR (TI "ICU") OR (TI "critical care") OR (TI "critical illness") OR (TI "critically ill") OR (AB intensive care unit*) OR (AB "intensive care") OR (AB "ICU") OR (AB "critical care") OR (AB "critical illness") OR (AB "critically ill") AND (MJ Patients) OR (MJ Family) OR (TI patient*) OR (TI "family") OR (TI "families") OR (TI family member*) OR (TI relative*) OR (AB patient*) OR (AB "family") OR (AB "families") OR (AB family member*) OR (AB relative*) AND (TI quality of death*) OR (TI quality of dying*) OR (TI good death*) OR (TI bad death*) OR (TI "QODD") OR (TI "GDI") OR (TI "CES") OR (AB quality of death*) OR (AB quality of dying*) OR (AB good death*) OR (AB bad death*) OR (AB "QODD") OR (AB "GDI") OR (AB "CES")
Igaku Chuo Zasshi	ICU/TH OR critical care/TH OR critically ill/TH OR ICU/TA OR intensive care unit/TA OR intensive care/TA OR critical care/TA OR critically ill/TA OR serious illness/TA OR critical illness/TA AND Patients/TH OR Family/TH OR patient/TA OR family/TA OR family member/TA OR relative/TA OR significant other/TA OR key person/TA AND quality of death/TA OR good death/TA OR "QODD"/TA OR "GDI"/TA OR "Care Evaluation Scale"/TA NOT animals/TH NOT humans/TH
